# Moderation effects of loneliness between fatalism and wellbeing during the COVID-19 pandemic

**DOI:** 10.1038/s41598-023-31480-4

**Published:** 2023-03-18

**Authors:** Rafael Miranda Ayala, Cristina Torrelles-Nadal, Giancarlo Magro Lazo, Gemma Filella Guiu

**Affiliations:** 1 Universidad Continental, Av San Carlos 1980, Huancayo, 12000 Peru; 2 INEFC- University of Lleida, Partida La Caparrella, s/n, 25192 Lleida, Spain; 3 University of Lleida, Avinguda de L’estudi General nº4, 25001 Lleida, Spain; 4 Universidad Andres Bello, Santiago, Chile

**Keywords:** Psychology, Health care

## Abstract

The COVID-19 pandemic has put more than just our physical health at risk. Due to containment measures, people have become increasingly isolated and have drastically reduced their daily social interactions. Many studies have already shown the negative effects of these measures, including fatalism. However, research linking fatalism during COVID-19 to well-being indicators is still limited. The goal of this study is to examine the relationship between COVID-19-related fatalism and well-being indicators, as well as the role of loneliness in moderating this relationship. Data was collected from 1,036 adults in Peru through an online survey that included the Quality-of-Life Index, the Fatalism Facing COVID-19 Scale, the Loneliness Scale, and the Mood Assessment Scale. Three models were tested using linear regression and ordinary least squares with bias-corrected bootstrapping. The results indicate that fatalism has a negative impact on quality of life and a positive effect on negative affect, and loneliness moderates both relationships, supporting the conclusion that fatalism exacerbates the effect of well-being indicators and negative affect.

## Introduction

Since the outbreak of COVID-19 in China in December 2019, the virus has rapidly spread across the world, resulting in a global crisis^1^ and affecting people’s health in numerous ways^2^. To control the spread of the virus, local agencies and governments have implemented various restrictive measures, including varying levels of "stay-at-home" orders^1^. The impact of the virus and these measures has been profound, causing significant changes in people's daily lives, such as altered routines, social distancing, mask wearing, closure of educational and public facilities, confinement, and unemployment, as well as impacting their well-being^2–4^. Research has explored the impact of stay-at-home measures and social contact restrictions on well-being during the COVID-19 pandemic. Some studies have investigated the effect of staying connected on well-being^5^, while others have analyzed the relationship between lockdowns and well-being and mental health^6,7^. As with previous pandemics, the COVID-19 pandemic has resulted in a mental health crisis due to the rise of negative psychological effects^8,9^. This can lead to feelings of resignation or discouragement as people struggle to cope with daily life and may be tempted to abandon healthy behaviors and risk their safety in an effort to return to pre-pandemic normalcy^10^. Frustration with the "new normal" has led to passivity, resignation, and discouragement towards the situation, as well as fatalism, which creates a sense of helplessness.

Fatalism refers to a resignation attitude accompanied by the belief that daily actions have no impact on outcomes or inevitable life events^2,10–13^. Scholars have found that people with fatalistic beliefs often experience increased levels of psychological distress, feelings of isolation, negative emotions, and decreased well-being and quality of life^2,10,12^. Fatalistic beliefs also serve as a mechanism for reducing fear and anxiety by allowing people to stop trying to control the uncontrollable and thus reducing the stress caused by uncertainty.

The COVID-19 pandemic has led people to believe that they are unable to influence the situation^10,11^. In addition, this belief has been associated with low levels of well-being^14^, subjective well-being (WHO-5^15^), satisfaction with life^12^; and quality of life, as well as less preventive efforts against COVID-19^2,10,11^.

Quality of life is understood as the convergence of several important elements for the individual (e.g., psychological well-being, emotional well-being, satisfaction with life)^16^. It is understood as the perception one person has of its quality of life, in a general view or in relation to specific domains^17^. Mezzich et al.^18^ created the Quality-of-Life Index, which is a multidimensional subjective frame of reference for assessing quality of life that comprises elements like well-being^16^). Regarding well-being, it is important to understand it as a complex concept that is composed of remembered well-being and experienced well-being^19^. Remembered well-being is considered a cognitive component that is based on memories and judgments about people’s live. This component usually comprises life satisfaction and quality of life^20^. In turn, experienced well-being corresponds to all the appraisals that people make about affective states and feelings in real time, without considering memory. This component is usually analyzed with positive and negative affects^19^.

The pandemic situation affected on quality of life of people^21^ and decrease their levels of wellbeing, because it generates an emotional and cognitive stress^22^. The measures to prevent COVID-19 evoked negative emotions and increased the feeling of loneliness^21^. According to Clair et al.^23^, p 2), the perception of loneliness is a personal measure of social isolation, which could be understood as “the inadequate quantity and/or quality of interactions with other people, including those interactions that occur at the individual, group or community level”. According to Mellor et al.^24^, loneliness is positively related to depression, suicidal ideation, and is implicated in different negative aspects of mental health. Loneliness and this social isolation due to confinement reduce satisfaction with life and well-being, while increasing psychological distress in different contexts during COVID 19 pandemic^23,24–30^.

According to several studies^25,31,32^, university students are prone to experiencing loneliness, which is exacerbated during the COVID-19 pandemic^33^. Loneliness leads to an increased perception of threat, causing those who feel lonely to have more negative feelings towards COVID-19^34^. Arslan^25^ also highlights that loneliness has a detrimental impact on well-being, and is linked to anxiety and depressive symptoms in university students. Research indicates that individuals with high levels of loneliness have lower well-being. Additionally, there is a strong correlation between fatalism and lower well-being and negative feelings. It is therefore believed that the effect of fatalism on well-being and emotions will be more pronounced among individuals with high levels of loneliness.

This assumption of loneliness as a moderator is supported in recent evidence that shows loneliness having an effect on quality of life (interacting with sex)^35^) and also acting as a conditional -in the form of social isolation due to covid 19- in positive and negative affect (Li et al. 2020).

Based on the literature noted above, this study has two objectives. First, to analyze the relationship between the fatalism associated with COVID-19 and indicators of well-being. Second, to determine the role of loneliness in this relationship. The relationship between fatalism and well-being indicators could be conditioned by the perception of loneliness. Therefore, the following hypotheses are proposed: (H1) Fatalism will have a negative relationship with quality of life in specific domains and a positive relationship with negative affect; (H2) Loneliness will moderate the relationship between fatalism and quality of life in specific domains; (H3) loneliness will moderate the relationship between fatalism and negative affect and (H4) loneliness will moderate the relationship between fatalism and positive affect. The variables of sex and age will be used as control variables in the hypothesis testing.

## Method

### Participants and procedure

The participants of the study are adults living in Peru. Data collection was open and online. In total, 1036 adults responded the survey. The mean age of participants was 22.08 years (*SD* = 3.27). Likewise, regarding the sex of participants, 63.7% (*N* = 658) were females and 36.3% (*N* = 375) were males. A web-based convenience sample was used. However over 1000 observations were used for adequate estimation of the bias of the convenience sample estimator^37^.

Participation was voluntary. An informed consent was attached to the survey. This document explained the objective and the voluntariness of the study. Once signed the informed consent, the participant could start the survey. The study was conducted according to the guidelines of the Declaration of Helsinki, and approved by the ethics committee of Universidad Continental (protocol number of the study’s ethical approval: N° 004–2020-CE-FH-UC).

The survey was administered using Google Forms. Data was collected during the final months of 2020 in 14 out of 25 regions in Peru. Non-probabilistic sampling, specifically the snowball technique, was used to select participants. Respondents were encouraged to share the survey with their family and work connections to increase the number of participants.

### Instruments

#### Quality of life

This scale adapted by Mezzich et al.^18^ assesses the quality of life of a person in terms of objective and subjective vital conditions. This scale measures 10 relevant aspects: psychological well-being, physical well-being, self-care, autonomous functioning, occupational functioning, interpersonal functioning, emotional and social support, community and services support, professional realization, spiritual satisfaction and an overall appraisal of quality of life. The scale has a response interval that ranges from 1 to 10, (1 = bad to 10 = excellent). High scores in this instrument means an overall optimal perception of the different domains through which a person could appraise his or her quality of life.

#### Fatalism about COVID-19

Scale developed by Mejía et al.^38^, measures perceptions or beliefs about potential COVID-19 transmission situations through seven items (e.g., I believe that I will be admitted to hospital due to some complication). The scale is rated using a 5-point Likert scale This scale is assessed through a 5-point Likert scale (1 = totally disagree to 5 = totally agree). High scores in this instrument means a fatalist perception in the face of COVID 19 infection which is characterized by perceived upcoming health, mental and social threats without or with a very limited inner locus of control.

#### Loneliness

This scale was based on De Jong Gierveld’ Loneliness Scale^39^. Spanish adaptation^40^, includes eleven items with three response categories (0 = No, 1 = More or less and 2 = Yes). The scale assesses five items in a positive way (e.g., “I can count with my friends every time I need it”) and six, in a negative way (e.g., “I miss the company of other people”). High scores in this instrument means the presence of loneliness.

#### Emotions

This indicator was assessed through the Scale for Mood Assessment^41^, which has 16 items to measure transient mood states. In this case, respondents were asked to evaluate these moods during the pandemic. The scale is Likert-type with 11 points (0 = nothing to 10 = a lot). Items start with the affirmation “I feel”, and continue with an adjective that represents either positive or negative mood (e.g., “I feel sad”, “I feel happy”). Positive moods represented by the scale are: joyful, happy, optimistic and cheerful, considering also the following negative moods: e.g., mad, upset, sad. These emotions were measured differently in this study. In the case of positive emotions subscale high scores means the propensity of positive affect even through challenges, on the other hand high scores in negative subscale high scores means the propensity of negative affect and experiencing the world in a negative way.

### Statistical analysis

First, data collected was analyzed using a descriptive analysis of means and standard deviation for each variable to be analyzed. Prior to conducting descriptive analysis, a confirmatory factor analysis (CFA) was performed for each proposed variable using AMOS v.22 software. The robust maximum likelihood estimator (MLR) was utilized in these calculations. In accordance with Hu and Bentler^42^, the fit of the model was evaluated using the Tucker–Lewis index (TLI) and comparative fit index (CFI), with values expected to be above 0.90, and the root mean square error of approximation (RMSEA), with a value expected to be below 0.08.

Complementarily, reliability was calculated for each indicator using Cronbach’s alpha and McDonald’s omega coefficient (Ω). For assessing the internal consistency of indicators, Kline’s criteria^43^ were followed, which consider a coefficient acceptable if higher than 0.66, and good from 0.80.

The linear regression models reported in the study considered well-being indicators such as quality of life and positive and negative emotions as dependent variables and three models were calculated. In the first model, sex and age were selected as control variables. The second model included the independent variables of fatalism about COVID-19 and loneliness. In the third model, the moderation variable of the relationship between loneliness and fatalism about COVID-19 was included.

The analysis of third model was conducted using the PROCESS command^44^ macro in SPSS v.23. This macro employs an ordinary least squares approach and a bias-corrected bootstrap method (with 5000 bootstrapped samples) to estimate the conditional (moderated) effect. To test the moderation model, variables were centered to the mean and interactions were created by calculating the product of both variables. The linear regression analysis incorporated two variables and the moderation variable. To determine significant interactions, a simple slope analysis was performed at low (− 1 SD), and high (+ 1 SD) levels of the moderator^45^. Hypothesis were tested based on confidence intervals, effect size and significant interactions (*p*-value < 0.05).

For this analysis, the simple moderation model 1 proposed by Preacher, Rucker and Hayes^46^ was employed. Mild outliers in the dataset were detected for the four variables that were explored, however these were not eliminated because they were less than 5%. For the construction of the interaction graphs, we used Dawson's Excel MACROS available at this link: http://www.jeremydawson.co.uk/slopes.htm. All of the data and measures are available to download in an open repository at: https://osf.io/2m45x/?view_only=10abfba7596f44189fd32a88075411a0 (the link was blinded for peer review purposes).

## Results

Table 1 represents the descriptive results for the indicators of this study as well validity and reliability indicators. As observed, the mean scores for positive emotions (*M* = 6.71) and quality of life (*M* = 7.63) present a mean above the median of the scale range assessed as opposed to negative emotions (*M* = 5.18), fatalism (*M* = 2.68) and loneliness (*M* = 0.58), whose values have a lower mean compared to the range median of these indicators. Regarding CFA analysis, good fit indicators presented are adequate according literature. As well, reliability indicators presented in Table 1 are higher than 0.70.

**Table 1:** Descriptive, validity and reliability analysis of study variables (N = 1036).

Variables	Range	Mean (*SD*)	Alpha	Omega	TLI	CFI	RMSEA
Quality of life	(1–10)	7.63 (1.46)	0.94	0.94	0.93	0.94	0.09
Negative emotions	(1–10)	5.18 (2.31)	0.98	0.96	0.91	0.93	0.09
Positive emotions	(1–10)	6.71 (1.85)	0.88	0.87	0.98	0.99	0.07
Fatalism	(1–5)	2.68 (0.80)	0.78	0.78	0.97	0.94	0.07
Loneliness	(1–3)	0.58 (0.28)	0.80	0.82	0.99	0.98	0.04

Prior to the linear regression and moderation analyses, a correlation analysis was performed between the variables in the hypothesis of this study. The results of Pearson correlations are presented in Table 2. As observed, all the relationships between variables were significant. (*p* < 0.01). However, based on the standard interpretation of effect size^47^, there is a strong association between quality of life and positive emotions (*r* = 0.54), while the associations between loneliness and quality of life (*r* = −0.49), loneliness and positive emotions (*r* = −0.38), loneliness and negative emotions (*r* = 0.39), fatalism and negative emotions (*r* = 0.30), and quality of life and positive emotions (*r* = −0.39) are moderate. The rest of associations show a weak level of association.

**Table 2:** Analysis of correlations between the variables of the model.

	1	2	3	4	5
1. Loneliness	–	0.23**	− 0.49**	− 0.38**	0.39**
2. Fatalism		–	− 0.26**	− 0.21**	0.30**
3. Quality of life			–	0.54**	− 0.39**
4.Positive emotions				–	− 0.16**
5. Negative emotions					–

**The correlation is significant at the 0.01 level (bilateral).

Table 3 presents the results of the analysis, including control variable regressions and considering quality of life as the dependent variable. Model 1 comprised the control variables of sex and age. For this model, only the age variable results (*β* = 0.03, *p* < 0.05). In turn, Model 2 employed the variables of the first model but also included the independent variables of fatalism about COVID-19 and loneliness separately. In the case of fatalism, *β* = −0.29 and *p* < 0.001 are reported, while for loneliness these values are *β* = −2.32, and *p* < 0.001.

**Table 3:** Linear regression and Hayes’ linear regression analyses considering loneliness a moderator (dependent variable = quality of life).

	Model 1				Model 2				Model 3			
	β	t	LLCI	ULCI	β	t	LLCI	ULCI	Β	t	LLCI	ULCI
Age	0.03	2.2	0.01	0.06	0.003	0.25	− 0.02	0.03	0.01	0.51	− 0.02	0.30
Sex	0.10	0.81	− 0.11	0.27	0.13	1.59	− 0.02	0.30	0.14	1.66	− 0.02	0.03
Fatalism					− 0.29	− 5.66	− 0.41	− 0.21	− 0.03	− 0.32	− 0.25	0.19
Loneliness					− 2.32	− 16.4	− 2.6	− 2.58	− 1.19	− 2.61	− 2.09	− 0.31
Fatalism x loneliness									− 0.43	− 2.61	− 0.76	− 0.11
R2	0.04				0.26				0.27			
F(df1, df2)	3.00(2,1029)				91.79 (4,1021)				75.2(5,1020)			

Model 3 conducted calculations through Hayes’ PROCESS macro in order to assess the moderating effect of loneliness in the relationship between fatalism about COVID-19 and the quality-of-life indicator. The combined effect of fatalism and loneliness was negative (*β* =  − 0.43, *p* < 0.01), and the regression coefficient for fatalism about COVID-19 indicator was not significant (*β* =  − 0.03, *p* > 0.05) but it was for loneliness (*β* =  − 1.19, *p* < 0.01).

The Table 4 slope test suggests that the relationship between fatalism and quality of life becomes stronger as reported loneliness increases. As can be seen, when the value of the loneliness moderator increases the effect on well-being is greater; for the case of low mild lof loneliness the reported effect is −0.16 on quality of life (*p* < 0.05); when the value of loneliness reaches a high level this effect of −0.40 on quality of life (*p* < 0.05).

**Table 4:** Slope test analysis of the moderation effects of the moderator (loneliness) and quality of life as dependent variable.

Moderation effects of moderator at M ± 1 SD (slope test)	Effect	SE	t	LLCI	ULCI
Loneliness Low -1 SD (− 0.28)	− 0.16	0.07	− 2.21	− 0.29	− 0.02
Loneliness Medium M (0.00)	− 0.28	0.05	− 5.57	− 0.38	− 0.18
Loneliness High + 1SD (0.28)	− 0.40	0.07	− 5.97	− 0.53	− 0.27

**Figure 1:**
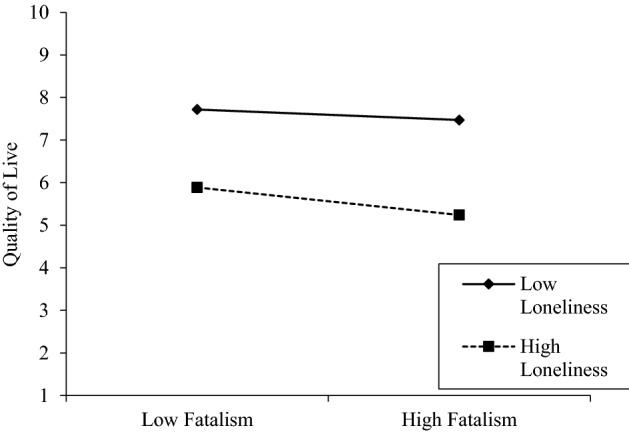
Moderation graph of the moderation for the buffer effect of experienced loneliness in the relationship between fatalism and life quality.

Likewise, the graphic analysis of the moderation in Fig. 1 indicates that the relationship between fatalism and quality of life presents a more pronounced slope with high levels of loneliness compared to the slope of this relationship with low values of loneliness.

Table 5 presents the regression coefficients of the variables considered in the hypotheses of this study about the negative emotions’ indicator. For Model 1, which considers only the control variables, both variables are significant. In the case of age, *β* = −0.07, *p* < 0.01, while for sex *β* = -0.50, *p* < 0.01. Model 2 adds the variables of fatalism about COVID-19 and loneliness. The regression coefficient for the age variable in this model is *β* = –0.04, *p* < 0.05, while for the sex variable is *β* = −0.56, *p* < 0.001. Regarding independent variables, fatalism about COVID-19 exhibits a significant relationship (*β* = 0.62, *p* < 0.001), as well as loneliness (*β* = 2.69, *p* < 0.001).

**Table 5:** Linear regression and Hayes’ linear regression analyses considering loneliness a moderator (Dependent variable: negative emotions).

VD = negative emotions	Model 1				Model 2				Model 3			
	β	t	LLCI	ULCI	β	t	LLCI	ULCI	Β	t	LLCI	ULCI
Age	− 0.07	− 3.31	− 0.11	− 0.03	− 0.04	0.02	− 0.07	0.01	− 0.03	− 1.77	− 0.07	0.01
Sex	− 0.50	− 3.43	− 0.77	− 0.19	− 0.56	− 4.2	− 0.80	− 0.28	− 0.53**	− 4.13	− 0.79	− 0.27
Fatalism					0.62	7.61	0.49	0.81	0.66***	7.68	0.50	0.82
Loneliness					2.69	11.6	2.16	3.07	2.58***	11.5	2.13	3.03
Fatalism x loneliness									− 0.53*	− 1.4	− 1.06	− 0.01
R2	0.02				0.21				0.21			
F(df1, df2)	12.13(2,1028)				68.80(4,1021)				55.48(5, 1020)			

Finally, Model 3, based on Hayes’ linear regression analysis, shows that most indicators are significant, except for the variable age (*β* =  − 0.03, *p* > 0.05). This model incorporates the interaction variable of fatalism about COVID-19 × loneliness which yields a significant coefficient of *β* = −0.53, *p* < 0.05.

Table 6 presents the moderating effects of the moderating variable, where the impact of fatalism about COVID-19 on experienced negative emotions is weakened with the increase of the loneliness indicator.

**Table 6:** Slope test corresponding to the moderating effects of the moderator (loneliness) and negative emotions as dependent variable.

Moderating effects of moderator at M ± 1 SD (slope test)	Effect	SE	t	LLCI	ULCI
Loneliness Low -1 SD (− 0.29)	0.81	0.12	7.03	0.59	1.04
Loneliness Medium M (.00)	0.66	0.08	8.12	0.50	0.82
Loneliness High + 1SD (.29)	0.51	0.11	4.74	0.30	0.72

**Figure 2:**
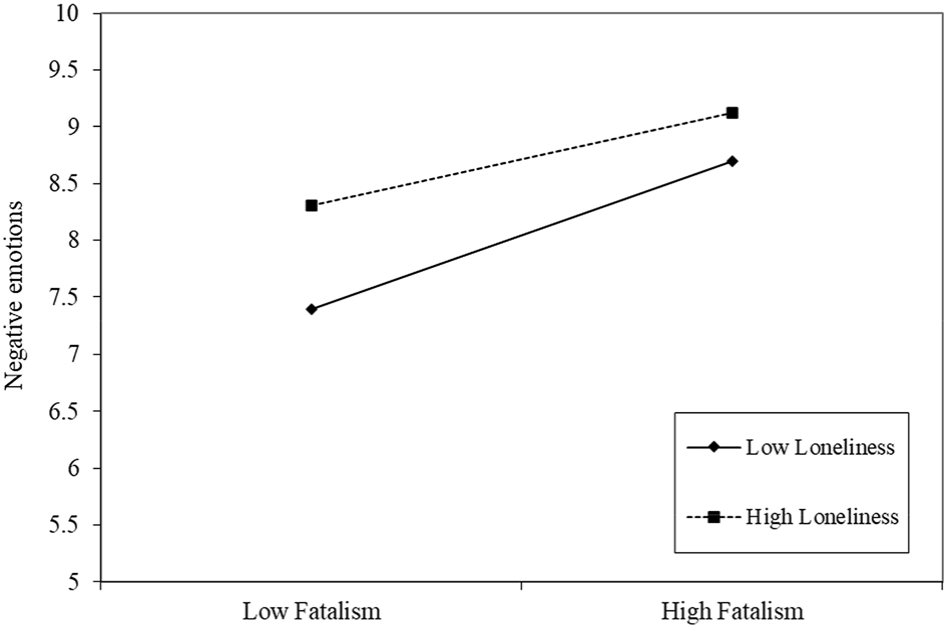
Moderation graph of the moderation for the buffer effect of experienced loneliness in the relationship between fatalism and negative emotions.

Finally, Fig. 2 shows the moderation effect of loneliness. The relationship between fatalism about COVID-19 and negative emotions has a steeper slope when lower levels of loneliness exist, compared with a straight line when there are higher levels of loneliness, for this case, the line is less steep.

The balance of affects presents both negative and positive emotions. Table 7 shows the results of the regression analyses when positive emotions were considered the dependent variable. When they were considered control variables (Model 1), both were not significant. Likewise, when incorporating the dependent variables separately into Model 1 (Model 2), the regression coefficient for the fatalism about COVID-19 indicator was negative and significant (*β* = −0.29, *p* < 0.001), as is the case of loneliness (*β* = −2.27, *p* < 0.001).

**Table 7:** Linear regression and Hayes’ linear regression analyses considering loneliness as a moderator (Dependent variable: positive emotions).

VD = positive emotions	Model 1				Model 2				Model 3			
	Β	T	LLCI	ULCI	β	t	LLCI	ULCI	β	t	LLCI	ULCI
Age	0.01	0.72	− 0.02	0.05	− 0.02	− 0.95	− 0.05	0.02	− 0.04	− 1.60	− 0.08	0.01
Sex	− 0.13	− 1.05	− 0.36	0.11	− 0.07	− 0.62	− 0.28	0.15	− 0.04	− 0.22	− 0.36	0.29
Fatalism					− 0.29	− 4.4	− 0.28	0.15	− 0.28	− 2.70	− 0.49	− 0.08
Loneliness					− 2.27	− 12	0.15	0.42	− 2.15	− 7.43	− 2.72	− 1.58
Fatalism x loneliness									− 0.03	− 0.09	− 0.73	0.67
R2	0.04				0.16				0.16			
F(df1, df2)	0.75(2,1028)				49.49(4,1021)				39.56(5, 1020)			

Finally, Model 3, based on Hayes analysis, indicates that interaction of independent variables was not significant; however, the regression coefficients were still significant for fatalism about COVID-19 and loneliness at the individual level. In the case loneliness about COVID-19, these were *β* = −2.15, *p* < 0.001, while the values were *β* = −0.28, *p* < 0.01 for fatalism about COVID-19.

## Discussion and conclusions

Descriptive results show average levels of fatalism COVID-19 infection in Peruvian adults. These findings are similar to those of a previous study conducted during the COVID-19 pandemic on the similar population^38^. According to the first hypothesis of this study, a significant relationship was observed between fatalism and indicators of well-being in both explored dimensions (quality of life and negative affects). These results are in line with previous studies, which established the relationship between both variables and found significant variations on cognitive and affective components of well-being (Diaz et al. 2015). Although some studies have begun to report these effects^2,10,11^, more studies are needed to fully understand this relationship, especially considering the delicate conditions experienced during the COVID-19 pandemic. Consequently, the results of this study constitute an important step to understand and measure specific well-being variations due to fatalism. Hopelessness has been previously associated with remembered well-being in depressed patients who perceived a deterioration of their quality of life^48^. It is possible that fatalism has followed a similar logic in influencing well-being. In addition, the threat of COVID-19 has been related to anxiety through negative affect as a moderator^49^. Fatalism refers to the belief that there is an imminent danger, which may cause psychological stress that manifests not as anxiety symptoms, but as a negative impact on overall well-being.

Regarding the second hypothesis, the results shows that loneliness moderates the effects of fatalism effect over indicators of perception about quality of life. Regarding the descriptive results, a strong negative relationship of these variables is observed in individuals with high levels of loneliness. The explanation for this result might be the change in the perception of quality of life’s domains and functioning due to confinement. Lonelier individuals—already affected by the negative consequences of loneliness associated with confinement—could perceive threats to economical and psychosocial well-being as more distressful than the direct impact of COVID-19 on physical health^34^. In other words, loneliness could moderate fatalism effects due the possibility of further disconnection. A study examined trajectories of loneliness during lockdown due to COVID-19 pandemic, finding that loneliness levels increased in the highest loneliness group in the first six weeks of lockdown, and decreased in the lowest loneliness group^33^. Individuals who are already lonely may be more greatly affected by confinement, leading to an increase in their feelings of loneliness and a greater disconnection from various aspects of their lives.

Concerning the third hypothesis, results shows that loneliness moderates the effect of fatalism over negative affect. On descriptive results, a strong positive relationship between these variables was observed in individuals with high levels of loneliness. Loneliness has been demonstrated to explain a significant variance of psychiatric symptoms in individuals during the COVID-19 pandemic^50^, therefore, one explanation for the moderation role of loneliness may be that fatalism in less lonely individuals could act as a mechanism for reducing fear and anxiety that has indirect effects on experienced well-being^2^. In lonelier individuals this same mechanism of resignation and helplessness could produce a further impact on this variable. Loneliness has been related to psychological functional affection and mainly to the possibility of developing depression^51^. This condition of loneliness as risk factor could explain this moderation effect by increasing the possibility of developing psychological distress symptoms associated with fatalism.

About our fourth hypothesis, results showed no statistical evidence of loneliness moderating the effect of fatalism over positive affect. Research^52^ shows that the variations of positive affect due to loneliness could depend on the level of negative affect. In our descriptive analysis it could be seen that teenagers showed a higher overall mean of positive affect than negative one. Even Though loneliness showed a significant interaction in positive affect at individual level, at interacting together with a variable that implies negative outcomes as fatalism it could depend on higher rates of negative affect in the sample to show a significant role. This is not the case of our research.

Finally, due to the limitations of this study, further studies with longitudinal designs are necessary to observe effects of loneliness interventions on the trajectories of fatalism and well-being.

Loneliness and perceived social isolation could be understood in a theoretical model of social cognition. From some authors^53^ the loneliness experience is equivalent to feeling unsafe and sets a tendency of hypervigilance. This unconscious surveillance for social threat could constitute a cognitive bias that alters executive functioning. One key element of this model is hypersensitivity to negative social information^54^. This could explain our results that showed loneliness as a moderator variable of the effects of Fatalism in wellbeing. During lockdown adolescents with higher levels of loneliness could have experienced higher affectation in their emotions and quality of life as a result of a diminished in their self regulation capacity by focusing unconsciously in the social threats about Covid 19. People with higher levels of loneliness may not handle confinement as well as people without this risk factor. Therefore, this group may need specific support on preventing further disconnection and further impact. In addition, the feeling of loneliness in the general population should be addressed, making use of specific programs to mitigate its effects^55^. Examples of such programs would be providing social support, promoting opportunities for interaction, or offering strategies to improve social skills.

## Data Availability

The datasets generated during and/or analysed during the current study are available in the OSF repository. The link was blinded for peer review purposes: https://osf.io/2m45x/?view_only=10abfba7596f44189fd32a88075411a0.
